# Vulnerability of locus coeruleus projections during aging

**DOI:** 10.1002/alz.092077

**Published:** 2025-01-09

**Authors:** Blanca Zufiria‐Gerboles, Daniel Veréb, Joana B Pereira

**Affiliations:** ^1^ Karolinska Institute, Stockholm Sweden; ^2^ Karolinska Institiute, Stockholm Sweden; ^3^ Neuro Division, Clinical Neurosciences Department, Karolinska Institute, Stockholm Sweden

## Abstract

**Background:**

The locus coeruleus (LC) is an important noradrenergic nucleus that supplies noradrenaline to the brain through extensive cortical and subcortical projections. Recent studies established that the LC is vulnerable to aging and common neurodegenerative disorders, including Alzheimer’s disease. However it is currently unclear how these projections differ between age groups and how they are affected by common age‐related comorbidities.

**Method:**

Here, we employ probabilistic tractography to characterize structural connectivity of the LC over aging using 3T diffusion‐weighted MRI in a population‐based cohort aged from 18 to 88 years of age (n=618). We assessed the relationship between age and connectivity, as well as common age‐related comorbidities, such as subjective memory problems, hypertension, diabetes, depression and sleep disturbances. Age‐related differences were assessed using Spearman’s rank correlation, whereas comorbidity‐related changes were investigated using partial least square analyses, corrected for age, sex and education.

**Result:**

The tractogram of the LC contained connections consistent with previous tractography and tracer studies. LC connectivity exhibited a marked decrease to a variety of brain areas including frontal and temporal regions, as well as the bilateral thalami and the left caudate (Spearman’s R < ‐0.2, p_FDR_<0.001). Participants with subjective memory problems exhibited increased LC connectivity to the ventral anterior cingulate cortex (p=0.0032). Participants with hypertension and diabetes exhibited decreased LC connectivity to the left middle frontal gyrus, the right amygdala and bilateral thalami (0.0001<p<0.014). Higher depression was associated with lower LC connectivity to the middle frontal, superior and inferior temporal gyri, whereas sleep problems were related to reduced temporal LC connectivity (p<0.05).

**Conclusion:**

These results provide an in vivo account of how the structural connectivity of the LC changes through aging and imply that LC connections are further affected by common age‐related disorders.

